# Wear, bone density, functional outcome and survival in vitamin E-incorporated polyethylene cups in reversed hybrid total hip arthroplasty: design of a randomized controlled trial

**DOI:** 10.1186/1471-2474-13-178

**Published:** 2012-09-20

**Authors:** Hugo C van der Veen, Inge van den Akker-Scheek, Sjoerd K Bulstra, Jos JAM van Raay

**Affiliations:** 1 Department of Orthopaedic Surgery, Martini Hospital, P.O. Box 30033, 9700, RM, Groningen, The Netherlands; 2 Department of Orthopaedic Surgery, University Medical Center Groningen, University of Groningen, P.O. Box 30.001, 9700, RB, Groningen, The Netherlands

## Abstract

**Background:**

Aseptic loosening of total hip arthroplasties is generally caused by periprosthetic bone resorption due to tissue reactions on polyethylene wear particles. *In vitro* testing of polyethylene cups incorporated with vitamin E shows increased wear resistance. The objective of this study is to compare vitamin E-stabilized highly cross-linked polyethylene with conventional cross-linked polyethylene in “reversed hybrid” total hip arthroplasties (cemented all-polyethylene cups combined with uncemented femoral stems). We hypothesize that the adjunction of vitamin E leads to a decrease in polyethylene wear in the long-term. We also expect changes in bone mineral density, less osteolysis, equal functional scores and increased implant survival in polyethylene cemented cups incorporated with vitamin E in the long-term.

**Design:**

A double-blinded randomized controlled trial will be conducted. Patients to be included are aged under 70, suffer from non-inflammatory degenerative joint disease of the hip and are scheduled for a primary total hip arthroplasty. The study group will receive a reversed hybrid total hip arthroplasty with a vitamin E-stabilized highly cross-linked polyethylene cemented cup. The control group will receive a reversed hybrid total hip arthroplasty with a conventional cross-linked polyethylene cemented cup. Radiological follow-up will be assessed at 6 weeks and at 1, 3, 5, 7 and 10 years postoperatively, to determine polyethylene wear and osteolysis. Patient-reported functional status (HOOS), physician-reported functional status (Harris Hip Score) and patients’ physical activity behavior (SQUASH) will also be assessed at these intervals. Acetabular bone mineral density will be assessed by dual energy X-ray absorptiometry (DEXA) at 6 weeks and at 1 year and 2 years postoperatively. Implant survival will be determined at 10 years postoperatively.

**Discussion:**

*In vitro* results of vitamin E-stabilized polyethylene are promising, showing increased wear resistance. However, controlled clinical follow-up data are not available at this moment.

This randomized controlled trial has been designed to determine wear, bone mineral density, functional outcome and survival in reversed hybrid total hip arthroplasty comparing cemented vitamin E-stabilized highly cross-linked polyethylene cups with cemented conventional cross-linked polyethylene cups.

**Trial registration:**

Dutch Trial Registry NTR3049

## Background

The most important factor for late implant failure in total hip arthroplasty is aseptic mechanical loosening of one or both components. The Nordic arthroplasty registries (Sweden, Norway and Denmark) show aseptic loosening as the main reason for revision in total hip arthroplasty (34.8-50.4%), followed by dislocation (23.4-33.5%) and infection (15.0-15.8%) [1]. Periprosthetic bone resorption, due to a cellular- and molecular-mediated foreign body reaction to polyethylene wear particles, is one of the main causes for this loosening [2,3].

In the 1950s and 1960s, total hip arthroplasty was performed using a metal-on-metal articulation [4,5]. In the 1970s, due to the success of Charnley’s low friction arthroplasty [6], metal-on-polyethylene articulations were increasingly applied. As polyethylene wear particles turned out to promote aseptic loosening, newer polyethylenes were developed to reduce wear. In the 1990s first-generation cross-linked ultra high molecular weight polyethylenes were introduced. This development led to a reduction in polyethylene wear. In looking for alternative bearing couples lacking the issue of polyethylene wear, the use of hard-on-hard bearings like metal-on-metal and ceramic-on-ceramic increased, especially in young active patients [7].

Hard-on-hard bearings also have shown disadvantages though. Although rare, ceramic articulations might lead to fracture of the ceramic bearing and produce squeaking noises [8]. Concerns remain over metal-on-metal articulations because of the metal ion release and subsequent elevated serum metal ion concentration [9]. Moreover, unpredictable local tissue reaction on metal particles can result in pseudo-tumor formation around the hip joint and perivascular lymphocytic infiltration because of a delayed-type hypersensitivity reaction (aseptic lymphocytic vasculitis associated lesion, ALVAL) [10]. A metal-on-polyethylene articulation seems therefore to be advocated above metal–on-metal articulation in total hip arthroplasty [11].

Wear characteristics of ultra-high molecular-weight polyethylene have been improved by the cross-linking technique [12,13]. A disadvantage is the increased oxidation and decreased fatigue strength of the polyethylene due to the irradiation, remelting and annealing process used in this technique. To prevent oxidation and increase mechanical strength, second-generation highly cross-linked polyethylene has been developed by incorporating vitamin E [14,15]. *In vitro* testing of these vitamin-E stabilized polyethylene cups shows good wear properties and improved mechanical and fatigue properties [16]. The influence of vitamin E-stabilized polyethylene cups on acetabular bone mineral density (BMD) is unknown. Hypothetically, this modified polyethylene leads to changes in acetabular bone mineral density due to its different mechanical properties.

The objective of this study is to compare vitamin E-stabilized highly cross-linked polyethylene with conventional cross-linked polyethylene in “reversed hybrid” total hip arthroplasties (cemented all-polyethylene cups combined with uncemented femoral stems). We hypothesize that the adjunction of vitamin E to polyethylene leads to a decrease in polyethylene wear in the long-term (primary outcome parameter). We also expect changes in BMD, less osteolysis, equal functional scores and increased implant survival in polyethylene incorporated with vitamin E (secondary outcome parameters) in the long term. The present paper reports on the design of the study.

## Methods

### Design

A randomized controlled trial will be conducted. Patients are randomized into one of the two treatment groups using concealed envelopes. Cluster randomization (per 10 patients) will be applied and the random allocator sequence will be computer-generated by an independent planner. Envelopes are opened one week before surgery. Patients are blinded for treatment. Only the surgeon and the primary investigator are aware of the allocation type. Follow-up is therefore done by an independent investigator, who is blinded for the treatment allocation. The study design, procedures and informed consent are approved by the local Medical Ethical Committee (registration number 2011-29). The trial is registered in the Netherlands Trial Registry (NTR3049). Guidelines of the Consort Statement are followed [17].

### Study population

The study will be conducted at the Department of Orthopaedic Surgery of the Martini Hospital, Groningen, the Netherlands. Patients to be included suffer from non-inflammatory degenerative joint disease of the hip and are scheduled for a primary unilateral total hip arthroplasty, and are aged under 70 years. Exclusion criteria are secondary osteoarthritis of the hip, (active) arthritis (e.g. rheumatic disease), peripheral neuropathy, history of CVA or cognitive impairment (e.g. dementia). Participation in the study is voluntary and informed consent is required. The inclusion period is planned from January 2012 to January 2014.

### Intervention

Both investigated groups will receive a reversed hybrid total hip arthroplasty. Gentamycine cement (Palacos®R + G, Heraeus) will be used for cup fixation, applying third-generation cementing techniques including vacuum mixing, pulsed lavage and pressurization. The same cementless 28-mm femoral component is used in both groups: a proximally plasma-sprayed porous coated titanium alloy (Ti6Al4V) stem (Mallory-Head®, Biomet) with a cobalt-chromium-molybdenum 28 mm femoral head.

One group will receive a cemented vitamin E-stabilized highly cross-linked polyethylene acetabular component (E1™ Muller cup, Exceed ABT Cemented Cup System, Biomet). The other group will receive a cemented conventional cross-linked polyethylene acetabular component without the adjunction of vitamin E (ArCom™ Muller cup, Exceed ABT Cemented Cup System, Biomet).

A posterolateral surgical approach in lateral decubitus position is used. Antibiotic prophylaxis with first-generation cephalosporin will be given preoperatively and during the first 24 hours intravenously.

### Follow-up

In this study the main outcome parameter is polyethylene wear (mm/y) 10 years postoperatively. Secondary outcome parameters are periacetabular bone mineral density, periprosthetic osteolysis, patient- and physician-reported outcome and implant survival (Table 1). Depending on the outcome parameters, measurements will take place preoperatively, 6 weeks, and 1, 3, 5, 7 and 10 years postoperatively.

**Table 1:** Secondary outcome parameters

	
1.	polyethylene wear at 1, 3, 5 and 7 years postoperatively
2.	relative decrease/increase in acetabular bone mineral density (BMD) at 1 and 2 years postoperatively
3.	acetabular and proximal femoral osteolytic changes at 1, 3, 5, 7 and 10 years postoperatively
4.	patient-reported functional outcome (HOOS and SQUASH) at 6 weeks and at 1, 3, 5, 7 and 10 years postoperatively
5.	physician-reported functional outcome (Harris Hip Score) at 6 weeks and at 1, 3, 5 and 10 years postoperatively
6.	survival (number of revisions) determined at 5 and 10 years postoperatively

Other study parameters: gender, age, *BMI*, severity of arthrosis, complications, size and inclination of the cup, surgical time and intraoperative blood loss.

#### Wear and osteolysis assessment

Wear will be determined on the routinely made conventional AP radiographs. Standard supine anteroposterior (AP) pelvic hip radiographs (with 115% magnification) will be taken. The X-rays at 6 weeks will serve as baseline, and will be compared 1, 3, 5, 7 and 10 years postoperatively. Wear will be assessed using the 2D measurement method as described by The et al. [18]. This method has been clinical validated by RSA and corrects for projection differences of the acetabular component. It has the advantage of approximating 3D-wear measurements without the need of lateral radiographs. Periacetabular and femoral radiolucencies will be assessed according to the three zones of De Lee and Charnley [19] and the femoral zones (especially zone 7) of Gruen et al. [20]. The scoring will be undertaken by an independent reviewer.

#### Bone densitometry

BMD measurements will be performed using a dual energy X-ray absorptiometry (DEXA) scanner (Hologic Inc., Bedford, Mass., USA) in order to calculate BMD changes in the pelvis as suggested by Wilkinson [21]. The contralateral normal hip will be scanned following a standard manufacturer's protocol to establish BMD in the femoral neck, trochanter, intertrochanteric, total hip and Ward's triangle sites.

#### Functional outcome

The Hip disability and Osteoarthritis Outcome Score (HOOS) will be used to assess patient-reported functional status, health-related quality of life and physical activity [22,23]. The short questionnaire to assess health-enhancing physical activity (SQUASH) will be used to determine habitual physical activity behavior [24]. Dutch versions of the HOOS and SQUASH will be used, as they are reliable and validated questionnaires for the Dutch THA population [25,26]. The Harris Hip Score will be used to assess physician-reported functional status, including range of motion [27,28].

### Sample size

It is our hypothesis that reversed hybrid total hip arthroplasties with vitamin E-stabilized polyethylene will show less polyethylene wear at 10 years compared to reversed hybrid total hip arthroplasty without the adjunction of vitamin E. In order to detect at least a clinically relevant difference in wear of 0.5 mm with a standard deviation of 0.07 mm, 63 patients are needed in each group (alpha 0.05, power 0.80). Based on previous studies we expect a drop-out rate of 15%. We therefore aim to include 75 patients in each group, thus 150 patients in total.

### Statistical analysis

#### Descriptive statistics

Descriptive statistics (means and standard deviations) will be used to describe the patient characteristics and outcome variables at the measurement points.

#### Univariate analysis

Student’s *T*-test will be used to determine if there is a difference in wear at 10 years postoperatively between the two groups. Differences in other outcome measures (osteolytic changes, bone mineral density, HOOS, Harris Hip score, SQUASH) between the two groups will also be analyzed with a student’s *T*-test (or non-parametric equivalent in case of a skewed distribution) at different measurement points.

#### Multivariate analysis

A GEE (Generalized Estimating Equation) analysis will be used to determine whether there is a difference on the primary and secondary outcome variables between the two groups over time, adjusted for relevant covariates. A p-value of < 0.05 is considered statistically significant.

## Discussion

As previously mentioned, *in vitro* results of vitamin E-incorporated polyethylene cups are promising, showing increased wear resistance and improved mechanical and fatigue properties. However, as yet no randomized controlled clinical data have been published on the mid- and long-term outcome of these modified polyethylene cups. This study primarily focuses on the outcome of wear in the long-term (10 years), comparing vitamin E-incorporated polyethylene with conventional polyethylene in reversed hybrid total hip arthroplasties. Short- and mid-term data will also be investigated in terms of periacetabular bone density and functional outcome.

To our knowledge, there are no randomized controlled trials that evaluate the incorporation of vitamin E in cemented polyethylene cups versus cemented polyethylene cups without the adjunction of vitamin E in total hip arthroplasty.

## Conclusion

This randomized controlled trial has been designed to determine wear, bone mineral density, functional outcome and survival in reversed hybrid total hip arthroplasty with vitamin E-stabilized highly cross-linked polyethylene versus reversed hybrid total hip arthroplasty with conventional cross-linked polyethylene.

## Competing interests

Biomet Netherlands finances the bone densitometry measurements. The authors will not receive any reimbursements, fees or salary for performing the study.

## Authors’ contributions

HCvdV designed the study and data collection protocols, coordinated the trial set-up, wrote the manuscript, and will analyze the data. IvdA-S assisted in the design and coordinated the trial set-up. SKB designed the study. JJAMvR initiated and designed the study. All authors read, edited and approved the final manuscript.

## Pre-publication history

The pre-publication history for this paper can be accessed here:

http://www.biomedcentral.com/1471-2474/13/178/prepub

## References

[B1] Havelin LI Fenstad AM Salomonsson R Mehnert F Furnes O Overgaard S Pedersen AB Herberts P Kärrholm J Garellick G The Nordic Arthroplasty Register Association: a unique collaboration between 3 national hip arthroplasty registries with 280,201 THRs Acta Orthop 2009 80 4 393 401 10.3109/17453670903039544 19513887 PMC2823198

[B2] Sochart DH Relationship of acetabular wear to osteolysis and loosening in total hip arthroplasty Clin Orthop Relat Res 1999 363 135 150 10379315

[B3] Purdue PE Koulouvaris P Nestor BJ Sculco TP The central role of wear debris in periprosthetic osteolysis HSS J 2006 2 2 102 113 10.1007/s11420-006-9003-6 18751821 PMC2488166

[B4] McKee GK Watson-Farrar J Replacement of arthritic hips by the McKee-Farrar prosthesis J Bone Joint Surg Br 1966 48 2 245 259 5937593

[B5] Ring PA Replacement of the hip joint Ann R Coll Surg Engl 1971 48 6 344 355 5089891 PMC2387876

[B6] Charnley J Arthroplasty of the hip A new operation. Lancet 1961 1 7187 1129 1132 10.1016/s0140-6736(61)92063-3 15898154

[B7] Manley MT Sutton K Bearings of the future for total hip arthroplasty J Arthroplasty 2008 23 7 Suppl 47 50 18701242 10.1016/j.arth.2008.06.008

[B8] Hannouche D Zaoui A Zadegan F Sedel L Nizard R Thirty years of experience with alumina-on-alumina bearings in total hip arthroplasty Int Orthop 2011 35 2 207 213 10.1007/s00264-010-1187-1 21191579 PMC3032121

[B9] Witzleb WC Ziegler J Krummenauer F Neumeister V Guenther KP Exposure to chromium, cobalt and molybdenum from metal-on-metal total hip replacement and hip resurfacing arthroplasty Acta Orthop 2006 77 5 697 705 10.1080/17453670610012863 17068698

[B10] Jacobs JJ Urban RM Hallab NJ Skipor AK Fischer A Wimmer MA Metal-on-metal bearing surfaces J Am Acad Orthop Surg 2009 17 2 69 76 19202120 10.5435/00124635-200902000-00003

[B11] Zijlstra WP Van Raay JJ Bulstra SK Deutman R No Superiority of Cemented Metal-on-Metal Over Metal-on-Polyethylene THA in a Randomized Controlled Trial at 10-Year Follow-up Orthopedics 2010 10 154 161 20349863 10.3928/01477447-20100129-19

[B12] Jacobs CA Christensen CP Greenwald AS McKellop H Clinical performance of highly cross-linked polyethylenes in total hip arthroplasty J Bone Joint Surg Am 2007 89 12 2779 2786 10.2106/JBJS.G.00043 18056513

[B13] Geerdink CH Grimm B Ramakrishnan R Rondhuis J Verburg AJ Tonino AJ Crosslinked polyethylene compared to conventional polyethylene in total hip replacement: pre-clinical evaluation, *in-vitro* testing and prospective clinical follow-up study Acta Orthop 2006 77 5 719 725 10.1080/17453670610012890 17068701

[B14] Wolf C Krivec T Blassnig J Lederer K Schneider W Examination of the suitability of alpha-tocopherol as a stabilizer for ultra-high molecular weight polyethylene used for articulating surfaces in joint endoprostheses J Mater Sci Mater Med 2002 13 2 185 189 10.1023/A:1013834113967 15348641

[B15] Wolf C Lederer K Bergmeister H Losert U Böck P Animal experiments with ultra-high molecular weight polyethylene (UHMW-PE) stabilised with alpha-tocopherol used for articulating surfaces in joint endoprostheses J Mater Sci Mater Med 2006 17 12 1341 1347 10.1007/s10856-006-0609-5 17143766

[B16] Oral E Christensen SD Malhi AS Wannomae KK Muratoglu OK Wear resistance and mechanical properties of highly cross-linked, ultrahigh-molecular weight polyethylene doped with vitamin E J Arthroplasty 2006 21 4 580 591 10.1016/j.arth.2005.07.009 16781413 PMC2716092

[B17] Moher D Schulz KF Altman DG The CONSORT statement: revised recommendations for improving the quality of reports of parallel-group randomised trials Lancet 2001 357 9263 1191 1194 10.1016/S0140-6736(00)04337-3 11323066

[B18] The B Flivik G Diercks RL Verdonschot N A new method to make 2-D wear measurements less sensitive to projection differences of cemented THAs Clin Orthop Relat Res 2008 466 3 684 690 10.1007/s11999-007-0077-3 18264857 PMC2505227

[B19] DeLee JG Charnley J Radiological demarcation of cemented sockets in total hip replacement Clin Orthop Relat Res 1976 121 20 32 991504

[B20] Gruen TA McNeice GM Amstutz HC "Modes of failure" of cemented stem-type femoral components: a radiographic analysis of loosening Clin Orthop Relat Res 1979 141 17 27 477100

[B21] Wilkinson JM Peel NF Elson RA Stockley I Eastell R Measuring bone mineral density of the pelvis and proximal femur after total hip arthroplasty J Bone Joint Surg Br 2001 83-B 283 288 11284582 10.1302/0301-620x.83b2.10562

[B22] Klassbo M Larsson E Mannevik E Hip disability and osteoarthritis outcome score. An extension of the Western Ontario and McMaster Universities Osteoarthritis Index. Scand J Rheumatol 2003 32 1 46 51 10.1080/03009740310000409 12635946

[B23] Nilsdotter AK Lohmander LS Klassbo M Roos EM Hip Disability and Osteoarthritis Outcome Score (HOOS)- Validity and responsiveness in total hip replacement BMC Musculoskelet Disord 2003 4 10 10.1186/1471-2474-4-10 12777182 PMC161815

[B24] Wendel-Vos GC Schuit AJ Saris WH Kromhout D Reproducibility and relative validity of the short questionnaire to assess health-enhancing physical activity J Clin Epidemiol 2003 56 12 1163 1169 10.1016/S0895-4356(03)00220-8 14680666

[B25] de Groot IB Reijman M Terwee CB Bierma-Zeinstra SM Favejee M Roos EM Verhaar JA Validation of the Dutch version of the Hip disability and Osteoarthritis Outcome Score Osteoarthr Cartil 2007 15 1 104 109 10.1016/j.joca.2006.06.014 16890460

[B26] Wagenmakers R van den Akker-Scheek I Groothoff JW Zijlstra W Bulstra SK Kootstra JW Wendel-Vos GC van Raaij JJ Stevens M Reliability and validity of the short questionnaire to assess health-enhancing physical activity (SQUASH) in patients after total hip arthroplasty BMC Musculoskelet Disord 2008 9 141 10.1186/1471-2474-9-141 18928545 PMC2576250

[B27] Harris WH Traumatic arthritis of the hip after dislocation and acetabular fractures: treatment by mold arthroplasty. An end-result study using a new method of result evaluation. J Bone Joint Surg Am 1969 51 4 737 755 5783851

[B28] Söderman P Malchau H Is the Harris Hip Score system useful to study the outcome of total hip replacement? Clin Orthop Rel Res 2001 384 189 197 10.1097/00003086-200103000-00022 11249165

